# Eye-Opener: A Case Report of Eyelid Taping as Presenting Symptom of Myasthenia Gravis

**DOI:** 10.21980/J8NW8G

**Published:** 2025-04-30

**Authors:** Mary G McGoldrick, Chirag N Shah

**Affiliations:** * Columbia University Irving Medical Center, Department of Emergency Medicine, New York, NY; ^ Rutgers Robert Wood Johnson Medical School, Department of Emergency Medicine, New Brunswick, NJ

## Abstract

Myasthenia gravis (MG) is an autoimmune disorder of the neuromuscular junction that can cause various symptoms provoking a visit to the emergency department (ED). In this case, we present a 54-year-old female who reported having her eyes “taped open” for the last two months. Her history and physical exam findings in the ED raised suspicion for MG. The patient was subsequently admitted and started on pyridostigmine. An elevated acetylcholinesterase receptor-binding antibody level confirmed the diagnosis of MG. This case report highlights the characteristic progressive weakness of facial muscles in MG, emphasizing the importance of early recognition of MG symptoms by emergency clinicians in order to initiate appropriate management and prevent respiratory compromise and morbidity.

**Topics:**

Neurology, neurologic exam, myasthenia gravis, emergency medicine.

**Figure:**
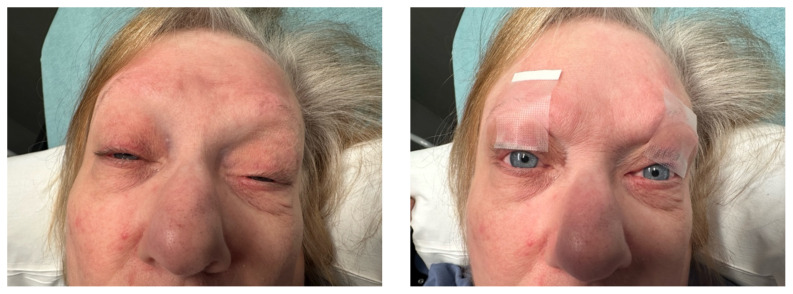
Video Link: https://youtu.be/i90aV1BL1c4

## Brief introduction

Myasthenia gravis (MG) is an auto-immune disorder of the neuromuscular junction, affecting an estimated range of 36,000 to 60,000 people in the United States per year.^1^ While the specific pathophysiology is dependent on the subtype of disease, MG ultimately decreases activity of acetylcholine (ACh) at the nicotinic acetylcholine receptors (n-AChR), resulting in impairment of neurotransmitter activity at the neuromuscular junction. Symptoms are characterized by fluctuating muscle weakness and fatigue. Ocular symptoms such as ptosis and diplopia are often recognized early, but bulbar weakness can be a less recognized initial presentation. Ocular weakness is the presenting symptom in up to 85% of patients, progressing to bulbar, axial, and limb involvement within two years in up to 50% of patients.^2^,^3^

Emergent presentations of MG, myasthenic crises, can be precipitated by several factors, such as infection, medication use, and environmental considerations.^4^ While uncommon, patients in frank myasthenic crisis can experience weakness of the diaphragm and respiratory muscles. These patients may present to the ED in respiratory failure, requiring emergent airway intervention and stabilization of breathing. Precursor symptoms such as those detailed above can be early indicators of a crisis.^5^

We present this case of characteristic progressive facial muscle weakness in the hopes of promoting prompt recognition of early MG symptoms by the emergency clinician, an essential step to appropriate management and prevention of respiratory compromise and morbidity. Written consent was obtained for use of the images for publication.

## Presenting concerns and clinical findings

Our patient was a 54-year-old female whose triage complaint was: “taping eyes open, last two months.” On evaluation, the patient described that over the past two months, her eyelids had gotten progressively weaker, requiring her to tape them open. As the day progressed, her symptoms would worsen. She noted blurred, double vision that was worsened when looking laterally in either direction. She also described that her tongue and jaw would, “wear out,” if she talked or chewed for too long during the day. While she was aware the symptoms were getting worse, she had been working with an optometrist that she knew, believing it to be an issue with her eyes. Her presentation to the ED was prompted by urging from family and growing concern for an underlying neurologic issue. The patient denied any difficulty swallowing, pooling of her secretions, or shortness of breath. She denied any numbness or weakness of her extremities, nor did she have any illnesses, or known exposures predating her symptoms, but did note she had her fourth COVID booster vaccine two weeks prior to onset. Past medical history included type 2 diabetes and leiomyosarcoma, status-post hysterectomy ten years prior. She was a nonsmoker, and did not use drugs or alcohol.

## Significant findings

Her vital signs were temperature 36.6° Celsius, heart rate 94 beats per minute, respiratory rate 16 breaths per minute, oxygen saturation 96% on room air, and blood pressure 150/79 millimeters of mercury (mmHg). Physical exam was significant for a very pleasant, well-appearing female in no acute distress, noted to have clear plastic tape attached to her bilateral eyelids and brows (Image 1). When the tape was removed, she had bilateral ptosis, more significantly in the left eye (Image 2). She had no conjunctival injection or pallor. Her airway was patent and protected. She had no neck masses or carotid bruits. Her heart and lung exams were normal, with no evident respiratory distress. Her neurologic exam was further significant for limited extra-ocular movement (EOM). Her most notable deficits were with lateral and upward gaze (Video 1) indicative of weakness at the muscles innervated by cranial nerves III and VI. Her pupillary response was symmetric and brisk bilaterally. She had no additional cranial nerve deficits, slurred speech, or asymmetry in her strength or sensation throughout.

## Patient course

The patient’s differential diagnosis in the ED consisted of myasthenia gravis, Miller-Fischer variant of Guillain-Barré, Lambert-Eaton myasthenic syndrome, infection, structural consideration such as aneurysm, mass effect, vitamin or electrolyte derangement or demyelinating disorder. She was screened with complete blood count (CBC), complete metabolic panel (CMP), erythrocyte sedimentation rate (ESR), C-reactive protein (CRP), magnesium level, folate and B12 level, as well as Lyme and syphilis screening. A computed tomography (CT) of the head as well as a computed tomography angiogram (CTA) of the head and neck were ordered. We also requested a negative inspiratory force at bedside with respiratory therapy, which was −36 centimeters of water (cm H_2_O).

All laboratory results obtained in the ED were within reference range. The patient’s CT and CTAs were significant only for an age-indeterminate lacunar infarct of the left thalamus. Neurology was consulted and performed an ice-pack test at bedside. The patient’s symptoms improved during the test, increasing clinical suspicion for MG. She was admitted to an internal medicine teaching service with neurology consult, pending a thyroid panel, magnetic resonance imaging (MRI) of the brain with and without contrast, CT of the chest to assess the lungs and thymus, and anti-acetylcholinesterase receptor (anti-AChR) testing.

During her hospital course, the patient was started on pyridostigmine, 30 milligrams (mg) three times daily, with improvement of symptoms. The medication was gradually titrated up to 60mg three times daily. Her inpatient results were significant for an unremarkable CT of the chest, redemonstration of the age-indeterminate lacunar infarct on MRI, and an ACh Receptor Binding Antibody level of 31.1 nanomoles per Liter (nmol/L) (reference range <0.02nmol/L), consistent with a diagnosis of MG. She was treated with intravenous immunoglobulin (IVIG) for five days. Her ptosis and bulbar weakness improved, and she was discharged on hospital day seven to follow up with outpatient neurology and neuro-ophthalmology. Per documentation from her follow up appointments, her pyridostigmine dose was increased to 180mg three times daily with improvement in her ptosis but continuing vertical diplopia. She was started on mycophenolate mofetil 500mg twice daily for these symptoms. To date, she has had no further hospitalizations for exacerbation.

## Discussion

Management of the patient with MG in the ED is typically centered on stabilizing and maintaining the airway in the setting of crisis, where signs of respiratory distress may be evident. In early or limited cases, the presentation may be more subtle. Fortunately, our patient’s disease did not appear to have progressed to involve her respiratory muscles. However, her complaint was not strictly ocular on further investigation of her symptoms. Knowing to screen for associated speech changes, weakness of the mouth, tongue, jaw, and swallowing is critical to identifying patients at risk for progression of their condition. The waxing and waning nature of symptoms can mask disease severity. A lack of rapid diagnostic testing makes MG difficult to definitively identify from other neuromuscular disorders in the emergent setting. Because MG is classified into ocular versus generalized, the ED clinician must understand the need for a complete assessment of patient’s ocular status as well as facial muscles, speech, and extremity strength.^6^

Patients presenting with evidence of respiratory compromise must be promptly recognized. Myasthenic crisis can occur in up to 20% of patients with MG and should be suspected in patients demonstrating signs of respiratory distress such as tachypnea, use of accessory muscles, or declining mental status. If performed, pulmonary function testing may demonstrate a diminished vital capacity (VC) or negative inspiratory force (NIF). Values below 1L or −30cm H_2_O, respectively, are indicative of crisis^7^ and should prompt clinicians to initiate supportive measures such as non-invasive positive pressure ventilation or endotracheal intubation.^8^ When intubating a patient with MG in the emergency department, clinicians must take special consideration when selecting a paralytic. Succinylcholine, a depolarizing paralytic that acts at the acetylcholine receptors, is ineffective due to lack of receptor functionality in patients with MG. Patients are also more sensitive to non-depolarizing paralytics and may require dose adjustment for rapid sequence intubation with agents like rocuronium. Variable response has been seen, with effective doses ranging from 0.3 milligrams per kilogram (mg/kg) to 1.2mg/kg.^9^

Additional management of myasthenia gravis involves removal of any potential exacerbating medications, such as beta-blockers, magnesium, fluoroquinolones, and aminoglycosides. In cases where MG is suspected, a trial of edrophonium resulting in transient improvement of muscle weakness can differentiate myasthenia from other potential causes. Mainstays of treatment include acetylcholinesterase inhibitors such as pyridostigmine (60–90mg by mouth every 4 hours or 2–3mg IV), corticosteroids (prednisone), and in severe, admitted cases intravenous immunoglobulin (IVIG) or plasma exchange. Our patient received pyridostigmine, steroids, and IVIG after discussion with the inpatient team due to concern for possible symptom progression.^10^, ^11^

This case highlights the importance of recognizing ocular weakness as a presenting symptom of MG in the ED. Prompt identification and initiation of appropriate treatment can prevent respiratory compromise and improve outcomes in these patients. Emergency physicians should maintain a high index of suspicion for MG in patients presenting with temporal weakness, especially involving the bulbar muscles, to provide the appropriate workup, intervention, and connection to specialist services.

## Supplementary Information






