# Supplementary figures and images for: Eye-Opener: A Case Report of Eyelid Taping as Presenting Symptom of Myasthenia Gravis
Source: J Educ Teach Emerg Med. 2025 Apr 30;10(2):V6–9. doi: 10.21980/J8NW8G (PMC12054071; doi:10.21980/J8NW8G)

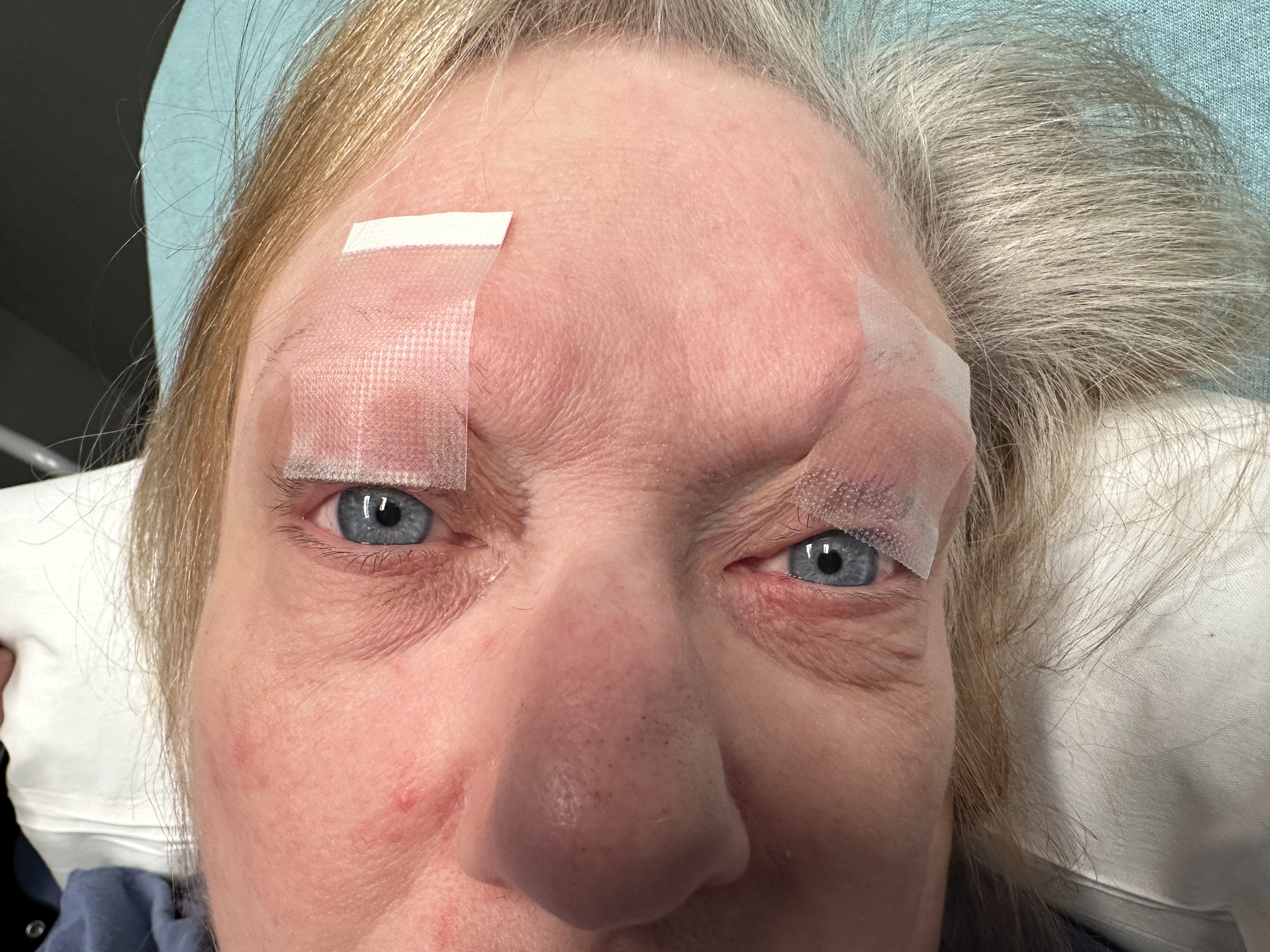

Supplement: Supplementary file 1 [file 10-2-V6-supp1.jpeg]

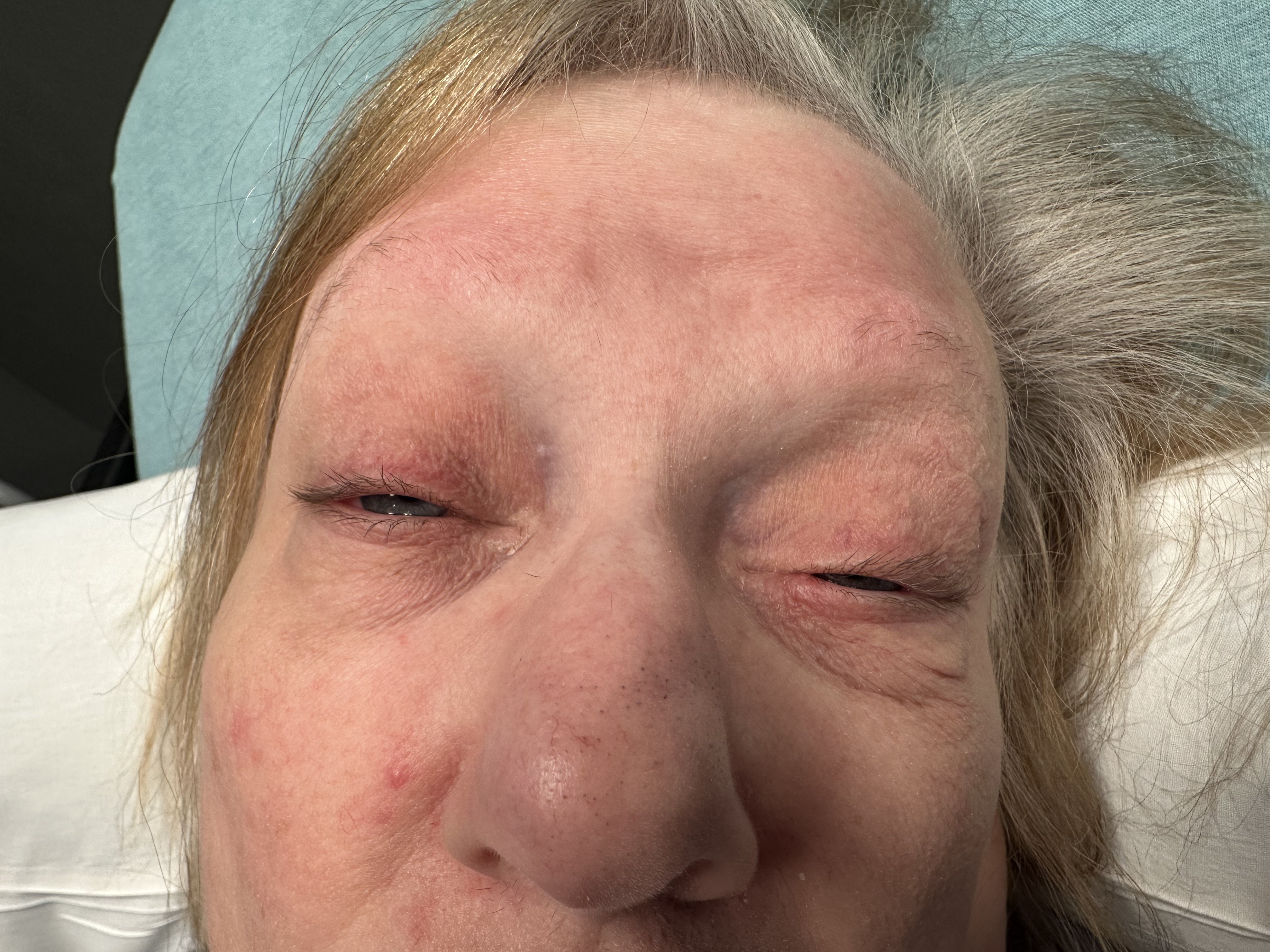

Supplement: Supplementary file 2 [file 10-2-V6-supp2.jpeg]
